# Effectiveness of steroid intra-articular injections on functioning in children and adolescents affected by juvenile idiopathic arthritis: a systematic review

**DOI:** 10.1007/s00431-025-06486-x

**Published:** 2025-10-20

**Authors:** Romina Gallizzi, Rosario Francesco Dipasquale, Alessia Mendicino, Martina Ferrillo, Umile Giuseppe Longo, Daniela Concolino, Antonio Ammendolia, Alessandro de Sire

**Affiliations:** 1 Department of Health Sciences, University of Catanzaro “Magna Graecia”, Catanzaro, 88100 Italy; 2 Dentistry, Department of Health Sciences, University of Catanzaro “Magna Graecia”, Catanzaro, 88100 Italy; 3 Fondazione Policlinico Universitario Campus Bio-Medico, Via Alvaro del Portillo 200, Rome, 00128 Italy; 4 Research Unit of Orthopaedic and Trauma Surgery, Department of Medicine and Surgery, Università Campus Bio-Medico Di Roma, Via Alvaro del Portillo 21, Rome, 00128 Italy; 5 Physical Medicine and Rehabilitation, Department of Medical and Surgical Sciences, University of Catanzaro “Magna Graecia”, Catanzaro, 88100 Italy; 6 Research Center On Musculoskeletal Health, MusculoSkeletalHealth@UMG, University of Catanzaro “Magna GraeciaUniversity of Catanzaro “Magna Graecia” , Catanzaro, 88100 Italy

**Keywords:** Juvenile idiopathic arthritis, Intra-articular injection, Steroid injection, Pain, Rehabilitation

## Abstract

**Introduction:**

Juvenile idiopathic arthritis (JIA) is the most common rheumatic disease of childhood, and steroid intra-articular injections are a fundamental treatment modality among local therapeutic interventions. The aim of this systemic review was to assess the scientific evidence on the effectiveness and safety of intra-articular corticosteroids (IACS) injections, focusing on a comparative examination of the different therapeutic options.

**Material and methods:**

PubMed, Scopus, and Web of Science were systematically searched from the inception until February 25, 2025, to identify observational studies presenting participants with a diagnosis of JIA, IACS injections for joints affected by arthritis as interventions, and clinical or radiological assessment of arthritis as outcomes.

**Conclusions:**

Findings from this systematic review suggested that IACS injections might be effective in improving arthritis in patients affected by JIA, with good evidence of safety. Moreover, the review underlines a higher efficacy of triamcinolone hexacetonide among corticosteroids used for injections. Further studies with a higher level of evidence and more representative samples should be conducted.

**Table:** 

**What is Known:** • Juvenile idiopathic arthritis is the most prevalent chronic rheumatic condition in childhood and represents a major cause of disability. • The management of juvenile idiopathic arthritis involves a variety of therapeutic modalities, among which intra-articular corticosteroid injections.	
**What is New:** • Intra-articular corticosteroid injections induce rapid symptom control and prolonged remission in a substantial proportion of patients. • Among different types of corticosteroids, triamcinolone hexacetonide is more effective in prolonging remission duration in JIA.

## Introduction

Juvenile idiopathic arthritis (JIA) is the most prevalent chronic rheumatic condition in childhood and represents a major cause of both short- and long-term disability. It is a systemic autoimmune disease characterized by persistent inflammation of the connective tissue, with onset before the age of 16 and a duration of at least six weeks [1, 2]. 

JIA has an estimated global incidence ranging between 1.6 and 23 cases per 100,000 children. The global pooled prevalence of JIA is estimated at 32.6 cases per 100,000 children [3, 4]. Prevalence data show greater variability than incidence, likely due to differences in access to pediatric rheumatology care, diagnostic capacity, and methodological inconsistencies among studies. Moreover, reliable prevalence data for non-Caucasian populations are lacking due to underrepresentation in epidemiological research [4].

The International League of Associations for Rheumatology (ILAR) classification system divides JIA into seven distinct subtypes. This framework was established to unify previous classification systems—those of the American College of Rheumatology (ACR), the European League Against Rheumatism (EULAR), and others—into a single, globally applicable model. The ILAR classification has become the gold standard for both clinical diagnosis and research [1, 2].

The management of JIA involves a variety of therapeutic modalities aimed at controlling inflammation, alleviating symptoms, preventing joint damage, and preserving long-term function. Treatment choice depends on disease subtype, severity, extent of joint involvement, and the presence of systemic manifestations.

Among local therapeutic interventions, intra-articular corticosteroid injections (IACIs) are a fundamental treatment modality, particularly for patients with monoarticular or oligoarticular disease. By delivering corticosteroids directly into the inflamed joint, IACIs achieve a potent and rapid anti-inflammatory effect with minimal systemic exposure [5].

The procedure can be performed under local or general anesthesia and is often facilitated by ultrasound guidance [6, 7].

In the treatment of children and adolescents, assessing skeletal maturation is crucial to define the most appropriate therapy [8–10].

Albeit IACIs are generally regarded as safe, both local and systemic adverse effects have been documented; common local reactions include transient post-injection pain or flare, typically resolving within 24 to 48 h. Cutaneous complications such as skin atrophy, hypopigmentation, and subcutaneous fat atrophy may occur, particularly following repeated injections [11, 12]. Systemic complications like transient hyperglycemia, HPA axis suppression, and allergic reactions are comparatively rare [13].

Triamcinolone acetonide (TA) and triamcinolone hexacetonide (TH) are synthetic corticosteroids extensively utilized in intra-articular injections. TA exhibits relatively higher aqueous solubility, which promotes faster dissolution and absorption within the synovial fluid [14]. This pharmacokinetic characteristic generally results in a shorter duration of anti-inflammatory activity, often requiring more frequent injections to sustain disease control. Conversely, triamcinolone hexacetonide is more lipophilic and less soluble, resulting in prolonged retention in the joint space [15]. This slower clearance facilitates a sustained release of the drug, offering a more durable therapeutic effect with reduced injection frequency.

Another corticosteroid used in the management of juvenile idiopathic arthritis is methylprednisolone acetate (MPA), although it is employed less frequently than TA and TH. Additionally, MPA is less lipophilic and has slightly lower anti-inflammatory potency compared to triamcinolone esters [11, 16].

The aim of this systemic review was to assess the scientific evidence on the effectiveness and safety of corticosteroids employed in intra-articular injections, focusing on a comparative examination of the different therapeutic options. The objective is to offer a comprehensive understanding of their clinical efficacy and potential adverse effects to support informed and effective treatment choices.

## Materials and methods

### Eligibility criteria

All observational studies were assessed for eligibility according to the following participants, intervention, comparison, and outcome model:

- Participants: children/adolescents with a diagnosis of JIA according to the ILAR classification of JIA;
- Intervention: intra-articular corticosteroid injections;
- Comparison: placebo or other approaches considered interventions, when available;
- Outcome: clinical or radiological assessment of arthritis (e.g., magnetic resonance imaging (MRI) findings, pain visual analog scale (VAS), function VAS, pain frequency, pain intensity, and pain index).

Exclusion criteria were as follows: (1) studies without measurable/objective outcome variables; (2) crossover study design; (3) studies involving animals; (4) studies written in a language different from English; (5) non-peer-reviewed studies (i.e., posters and conference abstracts); (6) three or more variables (such as participant or intervention groups/types); and (7) communications, correspondence, and editorials.

### Search strategy

PubMed, Scopus, and Web of Science databases were systematically searched for articles published from the inception until February 25, 2025, following the strategy described by Table 1. Furthermore, a manual search of the references of previous systematic reviews on a similar topic was conducted as well.

**Table 1:** Search strategy

Search strategy
PubMed(“juvenile idiopathic arthritis” OR “juvenile arthritis” OR “juvenile arthritis” OR “juvenile chronic arthritis”) AND (“joint disorder” OR “joint” OR “joint dysfunction” OR “articulation” OR “articulation disorder” OR “articulation dysfunction”) AND (“intra-articular injection” OR “intra-articular corticosteroid” OR “steroid injection” OR “joint injection” OR “joint corticosteroid” OR “triamcinolone” OR “acetonide” OR “hexacetonide” OR “triamcinolone acetonide” OR “triamcinolone hexacetonide”)
Scopus TITLE-ABS-KEY((((“juvenile idiopathic arthritis” OR “juvenile arthritis” OR “juvenile arthritis” OR “juvenile chronic arthritis”) AND (“joint disorder” OR “joint” OR “joint dysfunction” OR “articulation” OR “articulation disorder” OR “articulation dysfunction”) AND (“intra-articular injection” OR “intra-articular corticosteroid” OR “steroid injection” OR “joint injection” OR “joint corticosteroid” OR “triamcinolone” OR “acetonide” OR “hexacetonide” OR “triamcinolone acetonide” OR “triamcinolone hexacetonide”)))
Web of Science ((“juvenile idiopathic arthritis” OR “juvenile arthritis” OR “juvenile arthritis” OR “juvenile chronic arthritis”) AND (“joint disorder” OR “joint” OR “joint dysfunction” OR “articulation” OR “articulation disorder” OR “articulation dysfunction”) AND (“intra-articular injection” OR “intra-articular corticosteroid” OR “steroid injection” OR “joint injection” OR “joint corticosteroid” OR “triamcinolone” OR “acetonide” OR “hexacetonide” OR “triamcinolone acetonide” OR “triamcinolone hexacetonide”))

This systematic review with meta-analysis was conducted according to the guidance of Preferred Reporting Items for Systematic Reviews and Meta-Analyses (PRISMA) guidelines and the Cochrane Handbook for Systematic Reviews of Interventions. Systematic review protocol has been registered on the International Prospective Register of Systematic Reviews (PROSPERO) with number CRD42024519824.

### Data extraction

Two reviewers independently extracted data from included studies using a customized data extraction on a Microsoft Excel sheet. In case of disagreement, the consensus was achieved through a third reviewer.

The following data were extracted: (1) first author; (2) publication year; (3) journal; (4) study design; (5) demographic characteristics of study participants; (6) ILAR diagnosis; (7) systemic therapy; (8) type of intervention; (9) control group as comparison (placebo, or the approaches considered interventions), if available; (10) time points; and (11) main findings.

### Quality assessment

The risk of bias was assessed by two reviewers using the Joanna Briggs Institute (JBI) Critical Appraisal Checklist (Table 2). Any disagreement was discussed until a consensus was reached with a third reviewer.

**Table 2:** Joanna Briggs Institute Critical Appraisal Checklist for the studies included

Authors and year	Q1	Q2	Q3	Q4	Q5	Q6	Q7	Q8	Q9	Total score
Antonarakis et al., 2018	Y	Y	Y	Y	Y	U	Y	Y	Y	8
Arabshahi et al., 2005	Y	N	N	N	Y	Y	N	Y	Y	5
Aydın et al., 2022	Y	N	N	N	Y	Y	N	Y	Y	5
Cahill et al., 2007	Y	N	Y	N	Y	N	Y	Y	N	5
Chamlati et al., 2020	Y	N	N	N	Y	N	Y	Y	N	4
Chun et al., 2022	Y	Y	Y	Y	Y	Y	Y	Y	Y	9
de Oliveira Sato et al., 2014	Y	N	N	N	Y	Y	N	Y	Y	5
Eberhard et al., 2004	Y	Y	Y	Y	Y	Y	Y	Y	Y	9
Eberhard et al., 2012	Y	Y	Y	Y	Y	Y	Y	Y	Y	9
Habibi et al., 2012	Y	N	N	N	Y	Y	N	Y	Y	5
Harhay et al., 2021	Y	Y	Y	Y	Y	Y	Y	Y	Y	9
Lanni et al., 2011	Y	N	N	N	Y	Y	N	Y	Y	5
Leow et al., 2014	Y	N	N	N	Y	Y	N	Y	Y	5
Neidel et al., 2002	Y	N	N	N	Y	Y	N	Y	Y	5
Papadopoulou et al., 2013	Y	Y	U	N	Y	Y	Y	Y	Y	6
Resnick et al., 2016	Y	N	N	N	Y	Y	N	Y	Y	5
Ringold et al., 2008	Y	N	N	N	Y	Y	N	Y	Y	5
Rubin et al., 2022	Y	Y	Y	Y	Y	Y	Y	Y	Y	9
Stoll et al., 2012	Y	Y	N	N	Y	N	Y	Y	Y	6
Sukharomana et al., 2023	Y	N	N	N	Y	Y	N	Y	Y	5
Yıldız et al., 2024	Y	N	N	N	Y	Y	N	Y	Y	5
Zajc Avramovič et al., 2024	Y	N	N	N	Y	Y	N	Y	Y	5
Zulian et al., 2003	Y	Y	Y	Y	Y	Y	Y	Y	Y	9
Zulian et al., 2004	Y	Y	Y	Y	Y	Y	Y	Y	Y	9

Legend: Q1 = Is it clear in the study what is the “cause” and what is the “effect” (i.e., there is no confusion about which variable comes first)? Q2 = Were the participants included in any comparisons similar?; Q3 = Were the participants included in any comparisons receiving similar treatment/care, other than the exposure or intervention of interest?; Q4 = Was there a control group?; Q5 = Were there multiple measurements of the outcome both pre and post the intervention/exposure?; Q6 = Was follow up complete and if not, were differences between groups in terms of their follow up adequately described and analyzed?; Q7 = Were the outcomes of participants included in any comparisons measured in the same way?; Q8 = Were outcomes measured in a reliable way?; Q9 = Was appropriate statistical analysis used? *N*, no; *Y*, yes; *U*, unclear; *N/A*, not applicable

## Results

### Study characteristics

**Fig. 1:**
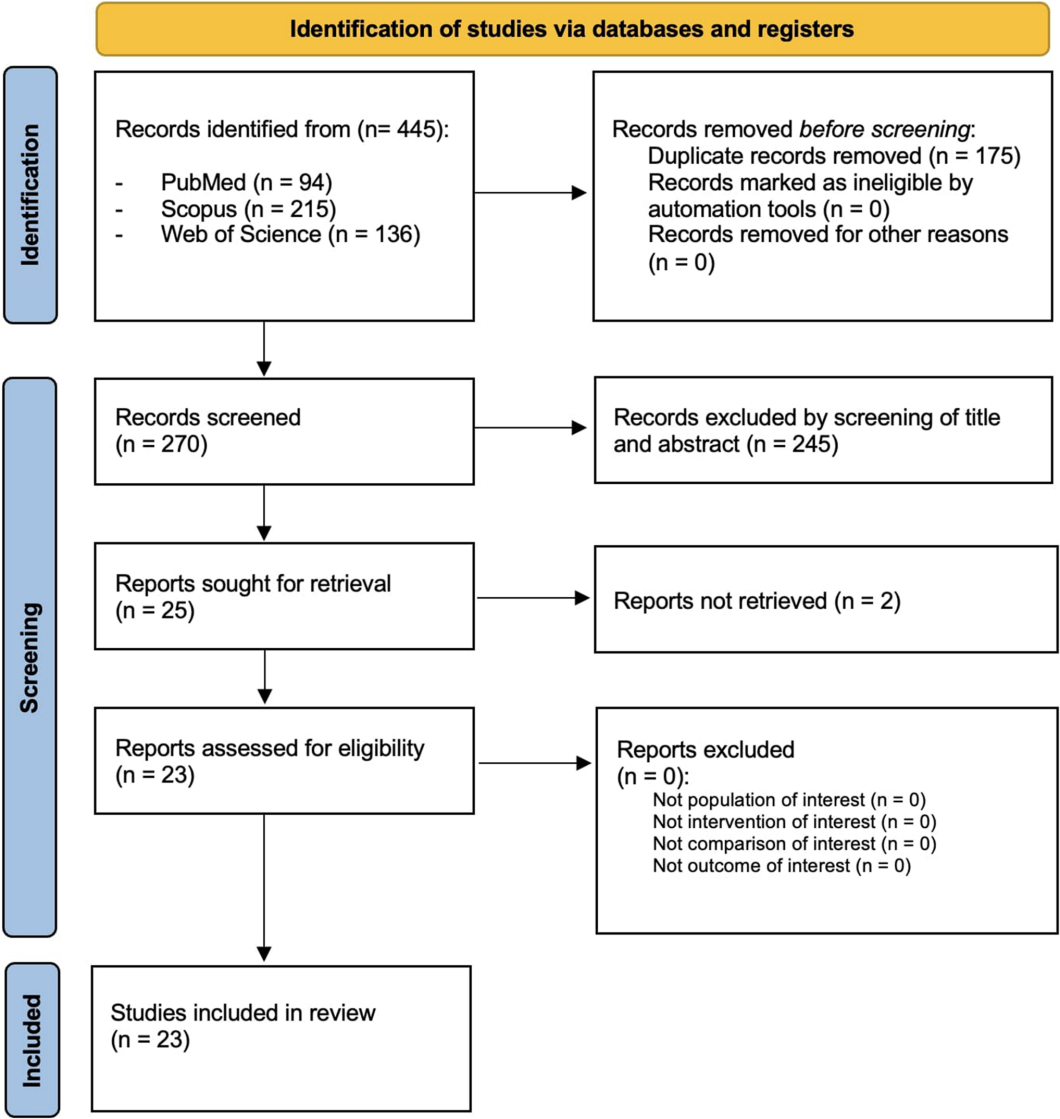
PRISMA 2020 flow diagram

The electronic search generated a total of 445 articles. After removing duplicates, 270 records were screened for title and abstract. Of these, 25 articles were assessed and 2 were excluded for the following reasons: not retrieved(*n* = 2). Thus, 23 studies were included in this systematic review, as reported by the 2020 PRISMA flow diagram (Fig. 1). The descriptive characteristics of the included studies are presented in Table 3. In terms of study design, twenty studies were retrospective longitudinal, three studies were prospective longitudinal, and one study was a randomized controlled trial. The publication year of the studies ranged between 2002 and 2024. The number of participants per study ranged from 10 to 440, with a total of 2158 participants, of which 469 were male, 1089 were female, and 570 not specified. The average ages of the study cohorts ranged from 2 to 18 years. All subjects had a diagnosis of JIA, performed according to ILAR classification of JIA. ILAR subgroup diagnosis and the administered systemic therapies are described in Table 3. Among the twenty-three studies considered, seven studies focused on a single joint (five studies on temporomandibular joint, one study on sacroiliac joint, one study on hip joint).

**Table 3:** Main characteristics of the studies included in the present systematic review

Authors, publication year, journal	Study design	Population (M/F)	Age (year/months)	ILAR diagnosis	Systemic therapy	Intervention	Comparison	Outcomes	Time points	Main findings in terms of primary outcome	Adverse events
Antonarakis et al., 2018, J Oral Maxillofac Sur	Prospective study	41 (M:5, F36)	13.6 ± 4.0 yr)	16 oligoarthritis, 12 rheumatoid factor-negative polyarthritis, 6 enthesitis-related arthritis, 3 undifferentiated arthritis, 2 rheumatoid factor-positive polyarthritis, 1 systemic arthritis, and 1 psoriatic	not specified	21 patients underwent TMJ arthrocentesis followed by intra-articular corticosteroid injection in at least one TMJ; 8 patients underwent arthrocentesis without the administration of corticosteroids;12 patients did not receive any local treatment and were observed as controls	Yes	To evaluate the change in the Helkimo anamnestic dysfunction index, which assesses patient-reported symptoms related to TMJ dysfunction	At baseline and 6 months after the intervention	The group that received TMJ lavage with IACS showed a statistically significant reduction in the Helkimo anamnestic dysfunction index scores at 6-month follow-up compared tom to the group that underwent lavage alone	No
Arabshahi et al., 2005, American college of Rheumatology	Retrospective study	23 (M:3/F:20)	4–10 y	Durban criteria	4 NSAIDs, 10 NSAIDs + MTX, 5 NSAIDs + MTX + antiTNFa	Sixteen of the 23 patients were injected with 1 cc (40 mg) triamcinolone acetonide in each of the involved TMJs. The remaining 7 patients were injected with 1 cc (20 mg) triamcinolone hexacetonide	No	To assess the effects of computed tomography (CT)–guided injection of corticosteroid into the temporomandibular joint (TMJ) in children with juvenile idiopathic arthritis (JIA) and clinical and magnetic resonance imaging (MRI) evidence of TMJ inflammation		Corticosteroid injections alleviated pain in more than two-thirds of symptomatic patients, one-third of these patients had persistence of effusions on followup MRI	Facial swelling indicative of Cushing’s syndrome occurred in two patients, persisting for 2 days in one case and for 2 weeks in the other.Following the injection, some patients reported a mild and temporary increase in TMJ pain
Aydın et al., 2022, Clinical Rheumatology	Retrospective study	134(M:47/F:87)	no	106 Oligoarticular, 6 extended oligoarticular, 10 polyarticular, 5 enthesitis-related, 3 undifferentiated, 2 psoriatic, 2 systemic	At the time of injection, all patients were receiving non-steroidal anti-inflammatory drugs, 74 patients were on methotrexate, and 14 patients were on biologics	IACIs with TH and methylprednisolone Triamcinolone hexacetonide (TH) at doses of 1 mg/kg (max 40 mg) for large joints (knee, shoulder, hip) and 0.5–1 mg/kg for medium joints (wrist, elbow, ankle, subtalar) was used. Methylprednisolone (MP) was used for small or difficult to access joints (finger and toe joints)	No	To investigate the outcome of IACI treatment	6 months	The median duration of remission without exacerbation of synovitis treated with IACI was 15 (range 1–64) months. The inactivity rate was 81%(109 patients) at the 6th month after the injection	Cutaneous hypopigmentation and subcutaneous atrophy were observed in two patients following the procedure
Chamlati et al., 2020, Pediatric rheumatology online journal	Retrospective study	43	13.8 y (8–18 y)	42 Juvenile Idiopathic Arthritis, 1 isolated sacroiliitis	Not specified	Sacroiliac joint injections of TH or TA	Yes	To review the feasibility and efficacy of image guided IACS in children	3 months (short-term) and 2 years (long-term) after the injection	Intra-articular injections provided short-term symptom relief, but in the longer term, most of the patients were maintained or escalated to systemic medical therapy within months to a year	No
Chun et al., 2022, BMC Rheumatology	Retrospective study	39	4.83 y (3.02, 7.67) for TH and 4.88 y (2.92, 10.65)	Oligoarticular, polyarticular, psoriatic	No	IACIs with TH and TA. TH was used at a dosage of 40 mg for large joints, 30 mg for the medium joints, 20 mg for the wrist and 4–6 mg for the small joints TA was injected at a dosage of 60–80 mg depending on the size of the patient for large joints, 40 mg for medium joints and 4–8 mg for small joints	Yes	To compare the duration of remission of IACIs with TH or TA among patients with JIA	10 years	The median time to relapse in months was higher for TH in comparison to TA both for all joints and knee only analysis (for all joints TH median = 18 and TA median = 4, for knee only TH median = 14 and TA median = 3)	IACSi typically causes mild and temporary side effects, with injection site discomfort being most common. Other effects include mood and sleep disturbances, appetite changes, menstrual irregularities, weight gain, and Cushingoid features, especially in young patients or those receiving multiple injections. Hypopigmentation and subcutaneous atrophy may occur but usually resolve over time. TH use was linked to greater post-injection discomfort but fewer systemic effects compared to TA, likely due to lower systemic absorption
de Oliveira Sato et al., 2014, Clinical and Experimental Rheumatology	Retrospective study	77(M:22/F:55)	no	44 persistent oligoarticular, 20 extended oligoarticular, 6 enthesitis related arthritis, 5 undifferentiated, 1 psoriatic arthritis, 1 polyarticular	no	IAC with TA and TH (1.5 (IQR 1.0–2.4) mg/kg of body weight.)	No	To estimate arthritis remission probability after single or repeated injections	4.3 years	Forty-four (57.1%) patients achieved inactive arthritis status, at some time point. Repeated injections resulted in remission off medication in only two (8.7%) cases, after the two injections session and one (6.3%) after three or more injection sessions. Repeated sessions varied from 1 to 5, including both repeated injection in the same joint or in newly affected joints. Taking into account all patients who were submitted to at least one session, the outcome at the last recorded visit was remission off medication in 20 (26%), remission on medication in 3 (3.9%), inactive disease in 15 (19.5%), disease-modifying anti-rheumatic drug prescription in 26 (33.8%), combina tion of disease-modifying anti-rheumatic drug and biological therapy prescription in 3 (3.9%), and active disease in 10 (13%)	Skin atrophy and hypopigmentation were documented in 8% of patients within the studied cohort
Eberhard et al., 2004, The Journal of Rheumatology	Retrospective study	85 (23 M, 62 F)	9.7 y ± 4.8 for TH and 8.8 ± 4.5 for TA	Oligoarticular, polyarticular, sistemic	In the TH group, 33 patients were taking a nonsteroidal antiinflammatory drug, 16 were taking methotrexate, 9 sulfasalazine, 3 were taking combination prednisone plus entanercept. In the TA group, 33 patients were taking a NSAID, 12 were taking MTX, 2 sulfasalazine, 4 etanercept, 2 prednisone, 2 leflunomide	IACIs with TH and TA TH was used in a dose of 40 mg for the knee, 30 mg for the ankle, 20 mg for the wrist and for the elbow. TA was used in a dose of 80 mg for the knee, 60 mg for the ankles, the wrist and the elbow	Yes	To compare patients with JIA injected with TH or TA with respect to time to relapse	2 weeks after joint injection and then every 3 months for a minimum of 15 months	The relapse time in the TH group was longer than that of the TA group (all joints: 10.36 ± 0.72 vs 8.45 ± 0.78 months; knee only: 11.11 ± 0.81 vs 7.95 ± 0.95 months) Both TH and TA have a prolonged duration of action; subjects injected with TH tend to relapse later than those injected with TA	No
Eberhard et al., 2012, The Journal of Rheumatology	Retrospective study	186 (41 M, 145 F)	9.7 y ± 4.8 for TH and 8.8 y ± 4.5 for TA	Oligoarticular, polyarticular, systemic	No	IACIs with TH and TA. Intra-articular TH was used in a dose of 40 mg for the knee, 30 mg for the ankle and elbow, and 20 mg for the wrist IA TA was used in a dose of 80 mg for the knee, 60 mg for the ankle and elbow, and 40 mg for the wrist	Yes	To evaluate whether the intraarticular dose of TH or TA influences time to relapse among patients with juvenile idiopathic arthritis	2 weeks after joint injections and then every 3 months for a minimum of 15 months	There was no statistically significant difference between high-dose (> 1 mg/kg) and low-dose (< 1 mg/kg) of IACS The average length of remission for TH use was 9.09 ± 3.51 months and for TA use 6.82 ± 3.44 months TH is more effective than TA with respect to time to relapse	No
Habibi et al., 2012, Rheumatology	Retrospective study	39(M:4/F:35)	12.25y(± 3.55, range 5–18)	3 systemic, 11 persistent oligoarticular, 9 extended oligoarticular, 12 rf-negative polyarticular, 1 rf-positive polyarticular, 1 psoriasic, 1 enthesitis-related	26 MTX, 9 TNFa, 1 anakinra	Triamcinolone hexacetonide [10 mg for children 1020 kg body weight (BW); 15 mg for 2040 kg BW and 20 mg for > 40 kg BW] was injected	No	To assess the safety and efficacy of US-guided IACS done by a paediatric rheumatologist into the TM joints in children with JIA	6–8 w	The most commonly documented physical examination findings at the first TMJ visit were decreased MIO (68%), deviation on opening (40%), and asymmetry of the mandible (12%). Following their initial IAS injection episode, 15 patients (60%) had no abnormality on TMJ examination documented by their physician, and 5 patients were noted to have persistent deviation or asymmetry of their mandible. At the final clinic visit of the study period, 18 patients (72%) had no documented abnormalities on TMJ examination. Of the 12 patients who had deviation or asymmetry of the mandible noted at the first clinic visit when their physician suspected TMJ disease, 2 had persistent deviation on TMJ opening noted at their final clinic visit	One case of scarring at the injection site
Harhay et al., 2021, Pediatric Rheumatology	Retrospective study	49(M:11/F:38)	< 16y	Oligoarticular only	No	IACIs with TA and TH. The doses used for TH were 1 mg/kg for large joints (knees and hips) and 0.5 mg/kg for smaller joints (ankles, wrists, and elbows) [8]. For TA, higher dose was used, ranging from 1.2–1.7 mg/kg for large joints	Yes	To compare response to treatment in children with oligo JIA who underwent therapy with intra-articular TA and TH injection	3–7 years	The results of the study are demonstrating the superior efficacy of TH over TA. After 6 months of IAC injection 73% of injected joints with TH group had sustained response compared to 50.8% in TA group. Twenty seven percent of joints injected with TH showed active arthritis 1–4 months after treatment compared to 49% of joints in the TA group showing no response. TH showed more efficacy despite using higher dose of TA in some of our patients The study examined a longer duration of follow up. Some patients are showing response for 3–7 years after a single joint injection particularly in the TH group. The duration of response for TH was 2.658 years and for TA was 1.70 years (p = 0.026) showing superior effect of TH for a longer duration.When examining the duration of response after IAC joint injection, it has been found that the duration of response for TH was significantly longer (2.66 years) than the response duration for TA which was 1.70 years (p = 0.026)	One case of subcutaneous atrophy was observed at the injection site
Lanni et al., 2011, Rheumatology	Retrospective study	440	2.2–10.8 y	20 Systemic arthritis, 70 RF-negative polyarthritis, 2 RF-positive plyarthritis, 171 persistent oligoarthritis, 147 extended oligoarthritis, 9 PsA, 8 enthesitis-related arthritis, 13 undifferentiated arthritis	126 ongoing or newly started MTX, 24 ongoing or newly started biologics, 37 ongoing or newly started systemic CS	IACIs with TH for large joints and methylprednisolone acetate (MPA) for small or difficult to access joints	No	To investigate the efficacy of IAC therapy in single and multiple joints in children with JIA	6 months	At 6 months after the IAC injection, remission of synovitis in all joints (injected and uninjected) was recorded in 65.1, 56.1 and 65.2% of patients who had received a single, double or multiple injection, respectively. An additional proportion of patients, which was sizeable among those injected in one or two joints (17.7 and 20.6%, respectively), but very small among those injected in three or more joints (1.7%), had remission of synovitis in all injected joints, but experienced flare of synovitis in one or more uninjected joints	Skin hypopigmentation and subcutaneous atrophy were the most common side effects, occurring in under 2% of patients, mainly at the wrist.Some patients experienced facial flushing 24–48 h post-procedure
Leow et al., 2014, Singapore Med J	Retrospective study	10 (M:6/F:4)	From 4 to 19 y	3 Oligoarticular, 2 Polyarticular RF-positive, 1 RF-negative, 1 Systemic-onset, 3 Enthesitis-related	Yes	26 Intra-articular injections (8 to knee joints, 7 to ankle joints, 4 to wrist joints and 2 to foot joints) of triamcinolone hexacetonide were administered to juvenile idiopathic arthritis patients who were receiving systemic treatment	No	To assess clinical efficacy and safety of IACS in juvenile idiopathic arthritis patients	At 3–6 months after injection	Out of 26 intra-articular glucocorticoid injections administered to patients with juvenile idiopathic arthritis, 18 joints demonstrated clinical improvement lasting from 2 to over 6 months. The mean clinical score improvement was 2.62 points (out of 15). Some joints required escalation of systemic therapy, particularly in oligoarticular JIA patients who did not receive increased systemic treatment at the time of injection. Most injections (21 of 26) did not require repeat administration within a few months; repeat injections occurred at intervals ranging from 4 to 9 months	Out of 22 injections with follow-up, 3 showed cutaneous adverse effects within six months: 1 case of subcutaneous atrophy (wrist) and 2 cases of hypopigmented macules (ankle, first metatarsophalangeal joint)
Neidel et al., 2002, Arthritis Rheum	Prospective study	48	10 y (2–17)	18 oligoarticular, 22 rf-negative polyarticular, 2 rf-positive polyarticular, 6 systemic	The following systemic medications were taken by the patients at that time (multiple entries were possible): NSAIDs (naproxen or indomethacin; 31 patients), methotrexate (24 patients), corticosteroids (20 patients), azathioprine (3 patients), sulfasalazine (2 patients), and auranofin (1 patient). Each child took at least one of these medications. During the study, NSAIDs were discontinued in 6 patients and started in 3 patients, methotrexate was discontinued in 5 patients and started in 4 patients, and sulfasalazine was started in 4 patients. At the end of the study, 3 children were not taking any medication because their disease was in full remission	IAC of TH (1 mg TH/kg body weight/hip joint, but not exceeding 40 mg/joint)	No	To study the efficacy and safety of intraarticular triamcinolone hexacetonide (TH) for the treatment of coxitis in patients with juvenile rheumatoid arthritis	2 y	In 39 of 67 hip joints (58%), remission of the coxitis for a period of 2 years was obtained through a single administration of IATH, while another 12 hip joints showed remission of coxitis after repeated TH injections (total remission rate 76%)	No
Papadopoulou et al., 2013, Arthritis Care & Research	Retrospective study	220 (45 M,175 F)	3.7 y (2.3–9.1)	5 systemic arthritis, 82 RF-negative polyarthritis, 4 RF-positive polyarthritis, 34 persistent oligoarthritis, 75 extended oligoarthritis, 1 psoriatic arthritis, 4 enthesitis-related arthritis, 5 undifferentiated arthritis	49 ongoing methotrexate,76 newly started methotrexate,21 ongoing or newly started biologic agents (13 etanercept, 4 anakinra, 2 adalimumab, 1 infliximab, and 1abatacept), 25 ongoing or newly started systemic corticosteroids	IACIs with TH for large joints and methylprednisolone acetate for small or difficult to access joints	Yes	To investigate the outcome of multiple IACS among patients with JIA	6 months	One-third of the patients sustained remission of synovitis in all injected joints after a median of 0.9 years from the injection. The cumulative probability of survival without synovitis flare was 50% at 1 year and 31.5% at 2 years	The most frequently observed adverse effect was skin hypopigmentation or subcutaneous atrophy localized to the injection site. This complication was documented in 0.9% of treated joints. Additionally, a subset of patients experienced flushing or erythema of the cheeks occurring 24 to 48 h post-injection
Resnick et al., 2016, J Oral Maxillofac Surg	Retrospective study	29(M: 5/F:24)	12.1 ± 1.9 y			Injection of 10 mg of triamcinolone hexacetonide	No	To quantify the effect of IACS on temporomandibular joint (TMJ) synovitis in children with juvenile idiopathic arthritis (JIA) using gadolinium-enhanced magnetic 75 resonance imaging (MRI)	22.9 ± 4.3 months	The ER decreased in all injected joints, with a mean reduction of 1.05. The post-IACS ER was less than the normal threshold (1.55) in 18% of the injected TMJs. IACS was associated with an elimination of pain in 89% of the subjects (P < 0.001) and in 88 augmentation of the MIO by 5.8 2.6 mm (P < 0.001)	No
Ringold et al., 2008, The Journal of Rheumatology	Retrospective study	25 (M: 4/F: 21)	Not specified	9 oligoarticular, 3 extended oligoarticular, 7 polyarticular RF-, 4 enthesitis-related, 2 psoriatic arthritis		The 25 patients underwent 74 IAS injections on 47 separate occasions. Each TMJ was injected with 0.5–1 ml triamcinolone acetonide (40 mg/ml) or triamcinolone hexacetonide (20 mg/ml).The type of corticosteroid used was determined by medication availability because triamcinolone hexacetonide was not available for roughly 1 year during the study period due to a sus- pension in manufacturing	No	To describe the clinical and radiographic outcomes in a series of patients with juvenile idiopathic arthritis (JIA) who underwent one or more IACS of the temporomandibular joint (TMJ) performed without imaging guidance	/	Ten patients (40%) had no documented TMJ complaints at the first clinic visit when their physician suspected TMJ involvement. Following their initial IACS, 18 patients (72%) had no documented TMJ complaints. At the final clinic visit of the study period, 21 patients (84%) had no documented TMJ complaints.The other 3 patients had new deviation documented over the course of the study duration One patient also underwent TMJ synovectomy for worsening clinical and radiographic findings during the data collection period. Moreover we found a mean increase in MIO of 6.9 mm (p = 0.002; 95% CI 3, 10.7). There was a mean increase in MIO of 3.8 mm following each IAS injection (p = 0.003; 95% CI 1.4, 6.2). Patients who underwent multiple IACS injections had a mean increase in MIO after first injection of 6.6 mm (p < 0.001; 95% CI 4.1, 9.1); however, the mean increase in MIO after subsequent injections was 0.4 mm (p = 0.8; 95% CI –3.5, 4.4)	Subcutaneous atrophy at the IAS injection site was observed in one patient following five right TMJ injections, ultimately necessitating surgical intervention for facial asymmetry, while two additional patients exhibited asymptomatic intra-articular calcifications on follow-up CT imaging
Rubin et al., 2022, Pediatric Rheumatology	Retrospective study	102(M:27/F:75)	No	85 persistent oligoarticular, 11 extended oligoarticular, 6 rf-negative polyarticular	-Patients with overall 28 joints were additionally treated with systemic MTX during the time of injection procedure. In 6/28 joints, the therapy was started 6–12 weeks prior the IACI -Patients with overall 6 joints were treated with anti TNFα during the time of IAJI procedure. in 3/6 joints the therapy was started 6–12 weeks prior the IACI	IACIs with TH and TA TH was used at a dose of 1 mg/kg (maximal dose of 40 mg) in knees, and 0.5 mg/kg (maximal dose of 20 mg) in ankles, elbows, and wrists. In smaller joints (wrist, midtarsal, and subtalar), 0.3 mg/kg (maximal dose of 10 mg) was injected. TA was used at a dose of 1–2 mg/kg (maximal dose of 80 mg) in knees, 0.5–1 mg/kg in ankles and el- bows (maximal dose of 40 mg), and 0.3–0.5 mg/kg (max- imal dose of 40 mg) in wrist, midtarsal, and subtalar joints	Yes	To compare the efficacy and safety of TA versus TH for JIA patients	21.9 (range 4–95) months	At 1 month: complete response 203 joints (96 TA, 107 TH), partial response 58 joints (32 TA, 26 TH), no response 28 joints (10 TA, 18 TH), relapse NA; At 3 months: complete response 200 joints (93 TA, 107 TH), partial response 26 joints (8 TA, 18 TH), no response 16 joints (6 TA, 10 TH), relapse 40 joints (27 TA, 13 TH)	Adverse events were uncommon and mild, with skin atrophy and hypopigmentation at the injection site occurring in only four joints (1.4%), evenly distributed between the two groups
Stoll et al., 2012, J Oral Maxillofac Surg	Retrospective study	63(M:20, F43)	Not specified	Not specified	5 None or NSAIDs alone, 2 CSs alone, 10 Conventional DMARDs alone ± CS, 5 Biologic DMARDs alone ± Cs, 41 Conventional and biologic DMARDs ± CS	Intra-articular corticosteroid injections (5–10 mg of triamcinolone hexacetonide) into the temporomandibular joints	No	To evaluate the improvement in maximal incisal opening (MIO) which reflects better jaw function	At baseline and from 1 month up to 2 years	The intra-articular corticosteroid injections led to a statistically significant improvement in maximal incisal opening (MIO). The mean MIO increased by approximately 3.2 mm after treatment	Three patients reported minor post-injection complications, including one case of localized swelling two days after the procedure, one case of fever two weeks post-procedure, and one case of skin hypopigmentation at the injection site observed 10 months after IACI
Sukharomana et al., 2023, Clinical Rheumatology	Retrospective study	45 (M:20/F:25)	10y (5.7–11.7)	19 Enthesitis-related arthritis, 6 persistent oligoarthritis, 6 extendet oligoarthritis, 3 RF-negative polyarthritis, 8 RF-positive polyarthritis, 3 undifferentiated	-In recents follow-up: 17 NSAIDs, 6 Prednisolone, 14 MTX, 12 MTX ans SSZ, 3 SSZ, 3 Biologics (etanercept) -At the time of joint injection in 174 of 177 (98.3%) joints, including NSAIDs (naproxen or ibuprofen), DMARDs (methotrexate, sulfasalazine, or combina- tion of both), and systemic corticosteroids (prednisolone). No concomitant biologics were used at the time of injection	IACIs with TA (Triamcinolone acetonide 1–2 mg/kg/dose (preparation 40 mg/ml) was used in large joints (maximum 80 mg) [7, 15], and TA 0.5 mg/kg/dose was used in medium joints, while the small joints of the hands and feet used TA 1–2 mg/dose (prepara- tion 10 mg/ml))	No	To explore the response to intraarticular triamcinolone acetonide (TA) injection in children with non-systemic juvenile idiopathic arthritis (JIA) and factors associated with time to arthritis flare	6, 12, 24 months	Of all 177 joints, a response to intraarticular TA injection at 6 months was observed in 118 (66.7%). Ninety- seven of 177 (54.8%) joints had arthritis flare after the procedure. The overall median time from intraarticular TA injection to arthritis flare was 12.65 months (95% CI 8.20–17.10 months). At 24 months after injection, 40.7% of all joints remained inactive	Local adverse effects following intra-articular TA injections were observed, including skin atrophy in 3 patients (1.1%), pigmentary alterations in 2 patients (1.7%), and radiographic evidence of calcification in 1 patient (0.6%)
Yıldız et al., 2024, Postgraduate Medicine	Retrospective study	225(M:122/F:103) of which 67IACI(M:27/F:40) and 158no IACI(M:76/F:82)	9.23 y (± 4.61; 0.08–18.08 y)	124.55%(n47) oligoarticular, 24.11% rf-negative polyarticular, 4.2% rf-positive polyarticular, 13.6% systemic, 6.3% psoriatic, 46.20% enthesitis-related, 8.3% undifferentiated	All patients who received IACI initially used NSAIDs as their first-line treatment. Among them, 88.1% (n = 59) received methotrexate, 47.8% (n = 32) received anti-TNF therapy (etanercept or adali- mumab), 17.9% (n = 12) received sulfasalazine, and 4 patients received tocilizumab treatment. Additionally, 35.8% of the patients received short-term bridging oral steroids during the course of treatment	IACI with TH TH was administered at a dose of 1 mg/kg to the knee and hip joints (maximum dose: 40 mg) and 0.5 mg/kg (maximum dose: 20 mg) to the ankle joints	No	To assess patients diagnosed with JIA who received intraarticular corticosteroid injections (IACS)	74.4(± 34,2) months	Recurrence of arthritis was not observed in the respective joints of 37 cases (59.7%). Moreover, among patients who received injections in multiple joints, arthritis recurred in only 3 cases, affecting only one joint in each case	IACI-related side effects occurred in 4.47% of patients, including subcutaneous atrophy in two cases and hypopigmentation at the injection site in one case. Subcutaneous atrophy resolved spontaneously within three years
Zajc Avramovič et al., 2024, Arthritis Research & Therapy	Retrospective study	109 (M:40/F:69)	8y (1.2y-18.3y)	80 Persistent oligoarthritis, 12 extended oligoarthritis, 5 psoriatic arthritis, 11 enthesitis related arthritis	No	IACIs with TH	No	To evaluate the long-term outcomes of patients with JIA oligoarthritis who received IACS as the first treatment for their disease	4.3 years (7 months-8.2 years)	After the first IAC 38.5% (42/109) did not require any further therapy and 14.7% (16/109) only required additional IAC	No
Zulian et al., 2003, Rheumatology	Prospective study	85 (19 M, 66 F)	58.5 months (13–144)	74 persistent oligoarticular, 11 extended oligoarticular	-Before the time of injection, 76 patients were receiving NSAIDs and 13 were receiving MTX -After the time of injection, 75 patients received NSAIDs and 12 received MTX	IACIs with TH and TA 1 mg/kg body weight (up to 40 mg) of intra-articular TH or TA	Yes	To compare the efficacy and safety of IACIs with TH and TA among patients with JIA	6, 12, 24 months	The rate of response, defined as the absence of synovitis or as a decrease in joint inflammation, was higher with TH than with TA (81.4% and 53.3% at 6 months, 67.1% and 43.3% at 12 months and 60% and 33.3% at 24 months) TH is more effective than TA in both short- and long-term follow-up	Skin atrophy at the injection site was observed in only two patients per group
Zulian et al.,2004, Rheumatology	Randomized controlled trial	37 (7 M 30 F)		22 persistent oligoarticular, 10 extended oligoarticular, 5 polyarticular	-Before the injection, 17 ongoing NSAIDs, 2 NSAIDs/CS, 6 NSAIDs/CS/MTX -After the injection, 20 received NSAIDs, 3 NSAIDs/CS,8 NSAIDs/CS/MTX	IACIs with TH and TA 1 mg/pro kg up to 40 mg of TH or 2 mg/kg up to 80 mg of TA	Yes	To compare the effects of intra-articular TA at a dose twice that of TH in symmetrically involved joints	3, 6, 9, 12, 18, 24 months	After the first injection, all joints pairs improved. In the follow-up period, 21 joints (53.8%) injected with TA relapsed compared with 6 (15.4%) injected with TH.Nine joint pairs were in full remission after 2 years. TH had a higher persisting or sustained response than TA (at 6 months 89.7% vs 61.5%, at 12 months 84.6% vs 48.7% and at 24 months 76.9% vs 38.5% respectively). The incidence rate of arthritis flare was higher for TA group (4.3/100 months vs 1.6–100 months) while TH group had a significantly greater probability to obtain remission (80% vs 47.5% after 12 months and 63.6% and 32.4% after 24 months)	Two patients, one in each group, developed skin atrophy at the injection site

### Intra-articular corticosteroid injections

All the studies evaluated the effectiveness of intra-articular corticosteroid (IACS) injections. In detail, Aydin et al. [17] conducted a retrospective study to investigate the outcome of intra-articular (IA) injections of triamcinolone hexacetonide (1 mg/kg for large joints and 0.5–1 mg/kg for medium joints) and methylprednisolone (for small or difficult to access joints) in a sample of 134 patients (47 males and 87 females, mean age at disease onset was 7.69 ± 4.62 years) over a period of at least 6 months. The authors showed an inactivity rate of 81% (109 patients) at the 6th month after the injection, with a median duration of remission without exacerbation of synovitis of 15 months. Lanni et al. [18] performed a retrospective study to investigate the efficacy of IACS using TH for large joints and MPA for small or difficult-to-access joints in a sample of 440 patients (mean age 2.2–10.8 years) evaluated at 6 months. Results showed remission of synovitis in all joints (injected and uninjected) recorded in 65.1, 56.1, and 65.2% of patients who received single, double, or multiple injections respectively. Leow et al. [19] in their retrospective study conducted on 10 patients (4 males and 10 females, mean age 4–9 years) underwent TH injections aimed to assess the clinical efficacy of IACS at 3–6 evaluated at 3–6 months after injection. Out of 26 IACS, 18 joints showed clinical improvement. Most injections (21 of 26) did not require repeat administration within a few months; repeat injection occurred at intervals ranging from 4 to 9 months. Papadopoulou et al. [20] conducted a retrospective study to investigate the outcome of IACS using TH for large joints and MPA for small or difficult-to-access joints in a sample of 220 patients (45 males and 175 females, mean age 3.7 years) followed for 6 months. Results showed one-third of the patients sustained remission of synovitis in all injected joints after 0.9 months after injection. Sukharomana et al. [21] performed a retrospective study to explore the response to IACS using TA (1–2 mg/kg in large joints, 0.5 mg/kg in medium joints, and 1.2 mg/dose for small joints) on a sample of 45 patients (20 males and 25 females, mean age 10 years) evaluated at 6, 12, and 24 months. Of all 177 joints injected, a response to intra-articular TA injection at 6 months was observed in 118 (66.7%). At 24 months after injection, 40.7% of all joints remained inactive. Yildiz et al. [22] conducted a retrospective study to assess the efficacy of IACS using TH (1 mg/kg for knee and hip joints and 0.5 mg/kg for ankle joint) on a sample of 225 patients (122 males and 103 females, mean age 9.23 years) followed for a mean period of 74.4 months. In this study, recurrence of arthritis was not observed in the respective joints of 37 cases (59.7%). Moreover, among patients who received injections in multiple joints, arthritis recurred in only 3 cases, affecting only one joint in each case. Zajc Avramovic et al. [23] led a retrospective study to evaluate the long-term outcomes of patients who received IACS using TH on a sample of 109 patients (40 males and 69 females, mean age 8 years) followed for a mean period of 4.3 years. After the first IACS, 38.5% (42/109) did not require any further therapy, and 14.7% (16/109) only required additional IACS.

### Intra-articular corticosteroid injection in specific joints

Seven studies have investigated the efficacy and safety of intra-articular corticosteroid injections administered to single joints, specifically the temporomandibular joint, the hip, and the sacroiliac.

Antonarakis et al. [24] in a prospective study compared the clinical and radiological outcomes of temporomandibular joint lavage alone versus lavage combined with intra-articular corticosteroid injection (triamcinolone hexacetonide). Patients receiving the combined treatment of lavage and corticosteroid demonstrated significantly greater pain relief and improved maximal mouth opening compared to those treated with lavage alone. Furthermore, magnetic resonance imaging showed a more substantial reduction in synovitis and joint effusion in the corticosteroid group.

Arabshahi et al. [25] conducted a retrospective observational study involving 258 patients to evaluate the efficacy and safety of temporomandibular joint (TMJ) corticosteroid injections, using TA or TH. Clinical outcomes were evaluated based on reductions in joint swelling, pain, and functional impairment, along with the duration of remission and the frequency of repeat injections. The results demonstrated that intra-articular corticosteroid injections effectively alleviated inflammatory symptoms, achieving a median remission period of approximately six months; however, resolution of pain does not always correspond to complete resolution of inflammatory signs observed on magnetic resonance imaging, such as joint effusion. Habibi et al. [26], in a retrospective cohort study, investigated the safety and efficacy of ultrasound-guided corticosteroid injections into the temporomandibular joints of children with active juvenile idiopathic arthritis.The intervention consisted of injections with triamcinolone hexacetonide, and outcomes were assessed both clinically and radiologically. Clinically, most patients experienced substantial improvement, with notable reductions in pain and enhanced mandibular function, particularly in maximal mouth opening. Imaging follow-up, including MRI and ultrasound, demonstrated a reduction in synovial hypertrophy and joint effusion, consistent with decreased inflammatory activity. The use of ultrasound allowed for accurate intra-articular needle placement, which likely contributed to the positive therapeutic response.Resnick et al. [27] evaluated the efficacy of intra-articular corticosteroid injections in the temporomandibular joints of pediatric patients with juvenile idiopathic arthritis. The study included 29 patients who received a total of 50 injections. The authors demonstrated a significant decrease in synovial enhancement ratio (ER) on MRI following intra-articular corticosteroid injections, accompanied by pain resolution in 89% of patients. Additionally, a notable improvement in mandibular function was observed, with maximal incisal opening (MIO) increasing by an average of 5.8 mm, highlighting both symptomatic relief and functional gains.Ringold et al. [28] conducted a retrospective cohort study assessing the safety and efficacy of intra-articular corticosteroid injections in TMJs. The study involved 25 patients who received a total of 74 injections administered by a single oral and maxillofacial surgeon without the use of imaging guidance. Outcomes measured included MIO, clinical symptoms, physical examination findings, and radiographic imaging. Results demonstrated a significant mean increase in MIO of 6.9 mm (P = 0.002). At baseline, 84% of patients exhibited radiographic signs of TMJ involvement. In their retrospective study, Stoll et al. [29] evaluated the safety and efficacy of IACIs in the temporomandibular joints. The study involved 63 patients who received a total of 137 IACIs administered by a single oral and maxillofacial surgeon without imaging guidance. The primary outcomes assessed were the safety of the procedure and efficacy as determined by changes in MIO. The results demonstrated a significant increase in MIO from 40.8 ± 0.93 mm to 43.5 ± 0.90 mm (P = 0.001). In a subset of 31 patients who underwent repeat MRI, 51% of TMJs showed evidence of improvement in arthritic changes, with 18% achieving complete resolution.The retrospective study of Chamlati et al. [30] aimed to evaluate the efficacy and safety of ultrasound-guided sacroiliac IACIs with TH or TA, focusing on treatment response and adverse events. The authors reported significant reductions in joint inflammation and pain, with many patients achieving remission following treatment. Neidel et al. [31] conducted a retrospective cohort study involving 50 patients, of whom 48 received a total of 67 injections across 67 hip joints. Remission of coxitis, defined as absence of pain and restored joint function, was achieved in 39 of 67 hips (58%) after a single injection. An additional 12 hips (18%) achieved remission following repeat injections, resulting in an overall remission rate of 76%.

### Intra-articular triamcinolone hexacetonide versus triamcinolone acetonide injections

Seven studies compared the effectiveness of IACS with triamcinolone hexacetonide versus triamcinolone acetonide.

Chun et al. [32] conducted a retrospective study on a sample of 39 patients (mean age of 4.83 years for TH and 4.88 years for TA) underwent IACIs with TH (40 mg for large joints, 30 mg for medium joints, 20 mg for wrist, and 4–6 mg for the small joints) and TA (60–80 mg depending on the size of the patient for large joints, 40 mg for medium joints, and 4–8 mg for small joints), and the authors showed a time to relapse in months higher for TH in comparison to TA for all joints and knee-only analysis (for all joints TH median = 18 and TA median = 4, for knee only TH median = 14 and TA median = 3).

Eberhard et al. [33] lead a retrospective study conducted on a sample of 85 patients (23 males and 62 females, mean age 9.7 for TH and 8.8 for TA) on medication (in the TH group, 33 patients were taking a nonsteroidal antiinflammatory drug, 16 were taking methotrexate, 9 sulfasalazine, 3 were taking combination prednisone plus etanercept. In the TA group, 33 patients were taking a nonsteroidal anti-inflammatory drug, 12 were taking methotrexate, 2 sulfasalazine, 4 etanercept, 2 prednisone, 2 leflunomide). The authors reported a longer relapse time in the TH group than that of the TA group evaluating all joints and knee-only analysis (all joints: 10.36 versus 8.45 months; knee only: 11.11 versus 7.95 months). Eberhard et al. [34] carried out a retrospective study conducted on a sample of 186 patients (41 males and 145 females, mean age 9.7 for TH and 8.8 for TA), and the authors reported a higher average length of remission for TH use (9.0 months) rather than TA use (6.82 months). Harhay et al. [35] conducted a retrospective study on a sample of 49 patients (11 males and 38 females, age < 16 years) underwent IACIs with TH (1 mg/kg for large joints and 0.5 mg/kg for smaller joints) and TA (1.2–1.7 mg/kg for large joints). The results demonstrated a superior efficacy of TH over TA. After 6 months of IACS, 73% of injected joints in the TH group had sustained response compared to 50.8% in the TA group. Twenty-seven percent of joints injected with TH showed active arthritis 1–4 months after treatment compared to 49% of joints in the TA group showing no response. The duration of response for TH was 2.658 years and for TA was 1.70 years. Rubin et al. [36] led a retrospective study conducted on a sample of 102 patients (27 males and 75 females) underwent IACIs with TH (1 mg/kg knees and 0.5 mg/kg in ankles, elbows and wrists; in smaller joints 0.3 mg/kg) and TA (1–2 mg/kg in knees, 0.5–1 mg/kg in ankles and elbows, 0.3–0.5 mg/kg in smaller joints). At 1 month and at 3 months, results showed higher complete response rates in joints injected with TH (107 at 1 month and 107 at 3 months) rather than TA (96 at 1 month and 93 at 3 months), with a higher relapse rate at 3 months for TA-injected joints (27) rather than TH-injected joints (13). Zulian et al. [37] conducted a prospective study on a sample of 85 patients (19 males and 66 females, mean age 58.5 months) underwent IACIs with TH and TA (1 mg/kg for both). The rate of response, defined as the absence of synovitis or as a decrease in joint inflammation, was higher with TH than with TA (81.4% and 53.3% at 6 months, 67.1% and 43.3% at 12 months and 60% and 33.3% at 24 months). In another study, Zulian et al.(2004) [38] carried out a randomized controlled trial performed on a sample of 37 patients (7 males and 30 females) treated with IACS using TH (1 mg/kg) and TA (2 mg/kg), in which the authors found a higher efficacy of TH than TA. In fact, in the follow-up period, 21 joints (53.8%) injected with TA relapsed compared with 6 (15.4%) injected with TH. Nine joint pairs were in full remission after 2 years. TH had a higher persisting or sustained response than TA (at 6 months 89.7% vs 61.5%, at 12 months 84.6% vs 48.7% and at 24 months 76.9% vs 38.5% respectively). This study is particularly notable for its use of symmetrical joint pairs, allowing for direct intra-patient comparison and effectively minimizing the need for an external control group. This methodological strength adds weight to the finding of superior and more sustained efficacy of triamcinolone hexacetonide (TH) over triamcinolone acetonide (TA).

### Single versus repeated IACIs

De Oliveira Sato et al. [39] evaluated the JIA remission after single or repeated IACIs, using TA and TH (1–2.4 mg/kg) in a sample of 77 patients (22 males and 55 females, mean age at first injection 10.1 years). The results showed that repeated injections lead to remission in only two cases (8.7%) after two injection sessions, and one (6.3%) after three or more injection sessions. Repeated sessions varied from 1 to 5, including both repeated injections in the same joint and in newly affected joints.

### Safety of IACSI

Out of 23 reviewed articles, 16 reported adverse effects related to IACIs. The side effects observed can be grouped as follows:

- Cutaneous and subcutaneous reactions
  The most commonly reported local adverse effects were skin hypopigmentation and subcutaneous atrophy [17–22], [28, 32, 35, 36, 38, 39]. Their incidence varied between less than 1% and approximately 8% of patients or treated joints, depending on the study. These effects were usually confined to the injection site, predominantly involving the wrist, ankle, and temporomandibular joint. In many cases, these reactions were mild, transient, and resolved spontaneously over time.
- Injection site discomfort
  Mild and temporary discomfort or pain at the injection site was reported [25, 32]. Some evidence suggested that injections with triamcinolone hexacetonide caused more post-injection discomfort than triamcinolone acetonide, potentially due to differences in systemic absorption profiles.
- Facial and systemic effects
  A small number of patients experienced facial swelling consistent with Cushingoid features, lasting from several days up to weeks [25, 32]. Additional systemic symptoms reported included disturbances in mood and sleep, appetite changes, menstrual irregularities, weight gain, and facial flushing or erythema occurring within 24 to 48 h after injection [18, 20, 29, 32].
- Other local effects
  Rare adverse events included skin scarring [26] and asymptomatic intra-articular calcifications [21, 28]. Notably, one case of subcutaneous atrophy required surgical intervention due to facial asymmetry following multiple TMJ injections [28].

## Discussion

This systemic review aimed to evaluate the current scientific evidence regarding the efficacy and safety of IACIs in children with JIA. The reviewed literature consistently highlights the clinical effectiveness of IACIs in the management of JIA, with particularly favorable outcomes observed in the oligoarticular subtype. IACIs have been shown to induce rapid symptom control and prolonged remission in a substantial proportion of patients, often after a single injection [17, 18, 23]. Several studies have reported marked clinical improvement, including reductions in joint swelling, pain, and stiffness, accompanied by decreases in inflammatory markers such as erythrocyte sedimentation rate (ESR) and C-reactive protein (CRP), thereby confirming the potent local anti-inflammatory efficacy of this therapeutic approach [17, 19–21, 23, 27]. Imaging techniques, particularly MRI, were employed in several studies to evaluate the anatomical and inflammatory status of the joints following intra-articular corticosteroid injections. Notably, radiologic evidence of ongoing synovitis or joint effusion did not always correspond with clinical improvement. In various cases, patients exhibited significant symptomatic relief despite persistent inflammatory findings on MRI, underscoring a potential dissociation between clinical remission and structural changes. These observations emphasize the need for a combined clinical and radiological approach when assessing therapeutic outcomes [25, 27, 28]. Several studies have investigated the efficacy of IACS in specific anatomical sites, including the TMJ, hip, and sacroiliac joint, with generally favorable outcomes. Multiple studies [24–28] demonstrated that IACIs significantly improved clinical symptoms such as pain and functional limitation, particularly maximal incisal opening (MIO). Nevertheless, despite these positive findings upon TMJ, the study led by Lochbühler et al. [40] introduced significant caution into the clinical opinion, demonstrating that intra-articular corticosteroids do not appear to prevent progressive osseous deformities of the TMJ or restore normal mandibular growth. As regards hip joint, in a retrospective study Neidel et al. [31] reported an overall remission rate of 76% following one or more corticosteroid injections. Importantly, femoral head necrosis (FHN) occurred exclusively in patients receiving concomitant long-term systemic corticosteroid therapy, while no cases were observed among those treated solely with intra-articular injections, suggesting a favorable safety profile for local therapy alone. Chamlati et al. [30] assessed ultrasound-guided IACS in the sacroiliac joints. The intervention led to significant reductions in joint inflammation and pain, with most patients achieving remission.

Seven studies comparing IACIs with TH versus TA consistently demonstrated superior efficacy of TH in prolonging remission duration in JIA. Median time to relapse was significantly longer with TH across various joints, including the knee, with reported remission durations ranging from approximately 9 to 18 months for TH compared to 3 to 8 months for TA [32, 33, 35–38]. A randomized controlled trial [38] further confirmed higher sustained remission rates with TH at 6, 12, and 24 months.

Regarding evaluation of single versus repeated IACIs indicated limited additional remission benefit from repeated injections, with low remission rates observed after multiple sessions [39].

Safety profiles of IACS were generally favorable, with adverse events predominantly mild and localized. Common side effects included transient skin hypopigmentation and subcutaneous atrophy at the injection site, occurring in up to 8% of cases. Injection site discomfort was frequently reported, with some evidence suggesting slightly greater post-injection pain following TH administration, potentially related to its pharmacokinetics. Rare systemic effects consistent with Cushing features and transient systemic symptoms were reported but remained infrequent. Severe complications, such as skin scarring or intra-articular calcifications, were rare, with isolated cases necessitating surgical intervention, primarily following multiple injections in sensitive sites like the temporomandibular joint, as reported in the scientific literature [41–47]. 

## Conclusion

In summary, the findings of the present systematic review showed that intra-articular corticosteroid injections had a robust efficacy and a favorable safety profile in managing juvenile idiopathic arthritis, particularly in oligoarticular subtypes. Triamcinolone hexacetonide consistently provides longer remission durations compared to triamcinolone acetonide, without significant differences related to dosing. While repeated injections offer limited additional benefit, adverse effects remain generally mild and localized, supporting the continued use of IACS as a valuable therapeutic option.

However, the current body of evidence is limited by the limited number of prospective, controlled clinical trials, that are crucial to directly compare the efficacy of various therapeutic strategies, especially intra-articular corticosteroids, or to evaluate long-term effects on mandibular development. Future research should also emphasize the use of validated outcome measures and incorporate imaging techniques such as ultrasound and/or MRI to ensure accurate and reproducible assessment of treatment response. Integration of clinical and imaging assessments is recommended to optimize patient management and monitor treatment response.

## Data Availability

The data that support the findings of this study are available from the corresponding author upon reasonable request.

## Abbreviations

ACR: American College of Rheumatology
CRP: C-reactive protein
ER: Enhancement ratio
ERA: Enthesitis-related arthritis
ESR: Erythrocyte sedimentation rate
EULAR: European League Against Rheumatism
FHN: Femoral head necrosis
ILAR: International League of Associations for Rheumatology
IA: Intra-articular
IACS: Intra-articular corticosteroid
IACIs: Intra-articular corticosteroid injections
JBI: Joanna Briggs Institute
JIA: Juvenile idiopathic arthritis
MPA: Methylprednisolone acetate
MIO: Maximal incisal opening
MRI: Magnetic resonance imaging
PRISMA: Preferred Reporting Items for Systematic Reviews and Meta-Analyses
PROSPERO: Prospective Register of Systematic Reviews
RF: Rheumatoid factor
TMJ: Temporomandibular joint
TA: Triamcinolone acetonide
TH: Triamcinolone hexacetonide
VAS: Visual analog scale
